# Novel Clustering Analysis Unveils Associations between Food Choices and Vascular Aging

**DOI:** 10.1093/geroni/igaf122.4165

**Published:** 2025-12-31

**Authors:** Chen Chen, Bangwei Chen, Li Luo, Shengyin Zeng, Jianguo Zhang, Tao Li

**Affiliations:** BGI Genomics, Shenzhen, Guangdong, China (People’s Republic); Tongji Hospital, Tongji Medical College, Huazhong University of Science and Technology, Wuhan, Hubei, China (People’s Republic); BGI Genomics, Shenzhen, Guangdong, China (People’s Republic); BGI Genomics, Shenzhen, Guangdong, China (People’s Republic); BGI Genomics, Shenzhen, Guangdong, China (People’s Republic); BGI Genomics, Shenzhen, Guangdong, China (People’s Republic)

## Abstract

Objective Vascular aging, marked by arterial stiffness, is a key risk factor for cardiovascular diseases (CVD) and a target for dietary interventions. However, dietary features influencing vascular aging are poorly understood. This study aimed to identify food choices linked to vascular aging in Chinese adults without CVD. Methods In this cross-sectional study of 1,155 adults, we collected dietary data (yes/no responses to 16 food items), sociodemographic characteristics, and body composition. Brachial-ankle pulse wave velocity (baPWV) was measured as the arterial stiffness indicator. Multiple correspondence analysis with hierarchical clustering on principal components were used to derive dietary clusters. Cramer’s V was calculated to measure the strength of association between variables and clusters. Generalized linear models were used to compare baPWV across clusters. Results Three distinct dietary clusters were identified, characterized by whole grain, root vegetables, smoked and salty foods (Cramer’s V > 0.5). Chinese-adapted Western diet (High smoked or salty foods intake) showed significantly higher baPWV compared to both Chinese-adapted Mediterranean diet (High whole grains or root vegetables intake, β: 30.52, 95% CI: 10.04-50.98) and conventional Chinese urban diet (balanced diet without favoritism, β: 22.91, 95% CI: 5.75-40.19). In contrast, there was no significant difference between Chinese-adapted Mediterranean diet and conventional Chinese urban diet. Conclusions We identified dietary groups based on simple food choice responses. The group characterized by preference for smoked or salty foods and low intake of whole grains or root vegetables, is associated with vascular aging. Keywords food choice; dietary pattern; vascular aging; arterial stiffness; multiple correspondence analysis

